# Design of Hierarchical Architected Lattices for Enhanced Energy Absorption

**DOI:** 10.3390/ma14185384

**Published:** 2021-09-17

**Authors:** Mohamad Al Nashar, Alok Sutradhar

**Affiliations:** Department of Mechanical and Aerospace Engineering, The Ohio State University, Columbus, OH 43210, USA; alnashar.1@osu.edu

**Keywords:** architected materials, lattices, energy absorption, simulation, hierarchical structures

## Abstract

Hierarchical lattices are structures composed of self-similar or dissimilar architected metamaterials that span multiple length scales. Hierarchical lattices have superior and tunable properties when compared to conventional lattices, and thus, open the door for a wide range of material property manipulation and optimization. Using finite element analysis, we investigate the energy absorption capabilities of 3D hierarchical lattices for various unit cells under low strain rates and loads. In this study, we use fused deposition modeling (FDM) 3D printing to fabricate a dog bone specimen and extract the mechanical properties of thermoplastic polyurethane (TPU) 85A with a hundred percent infill printed along the direction of tensile loading. With the numerical results, we observed that the energy absorption performance of the octet lattice can be enhanced four to five times by introducing a hierarchy in the structure. Conventional energy absorption structures such as foams and lattices have demonstrated their effectiveness and strengths; this research aims at expanding the design domain of energy absorption structures by exploiting 3D hierarchical lattices. The result of introducing a hierarchy to a lattice on the energy absorption performance is investigated by varying the hierarchical order from a first-order octet to a second-order octet. In addition, the effect of relative density on the energy absorption is isolated by creating a comparison between a first-order octet lattice with an equivalent relative density as a second-order octet lattice. The compression behaviors for the second order octet, dodecahedron, and truncated octahedron are studied. The effect of changing the cross-sectional geometry of the lattice members with respect to the energy absorption performance is investigated. Changing the orientation of the second-order cells from 0 to 45 degrees has a considerable impact on the force–displacement curve, providing a 20% increase in energy absorption for the second-order octet. Analytical solutions of the effective elasticity modulus for the first- and second-order octet lattices are compared to validate the simulations. The findings of this paper and the provided understanding will aid future works in lattice design optimization for energy absorption.

## 1. Introduction

Hierarchical cellular structures are ubiquitous in nature, featuring some unique mechanical properties. Inspired by these natural materials, cellular lattice structures such as honeycomb, sandwich cores, and foam have been created, and with the recent advent of additive manufacturing, newer topologies are being generated, and novel meta-materials are being formed. By creating hierarchical architected lattices at multiple scale lengths, mechanical properties and energy absorption can be tailored for high-performance applications. Energy absorption is an essential characteristic in the automotive and aerospace field, for example, for the crashworthiness of vehicles [1] or airplane fuselage [2]. Additionally, in the high-impact sports industry, efficient energy absorption is sought for designs of midsole and insole components in running shoes for comfort [3], and extreme sports helmet cushioning for safety [4,5].

Lattices have emerged as the main candidates for energy absorption structures and have been extensively studied [6,7,8]. Lattice unit cells are typically designed to harness instabilities and maximize buckling to increase energy absorption [9,10]. Additive manufacturing has opened up new possibilities for designing new lattice architecture. Additively manufactured lattices have demonstrated high energy absorption performance that scales exponentially with the relative density in the order of 2.5–3 [11]. Recently, there has been a rise in novel lattice design works. Hierarchical engineered structures have complex geometric topologies, and because of advancements in the additive manufacturing field, such designs are now attainable. For instance, a lot of new compositions and materials are now possible to 3D print in addition to the rise in numerous parametric optimization schemes that improve the quality of the additive manufacturing process [12,13]. Jin et al. [14] studied the dynamic behavior and energy absorption performance of various lattice configurations and reported that face-centered cubic (FCC) configurations such as an octet unit cell outperform other lattice configurations such as body-centered cubic (BCC), diamond lattice structure (Dfcc), and hexagonal diamond lattice structure (Dhex) in terms of specific strength, specific modulus, and energy absorption. Al-Saedi et al. demonstrated improvements in mechanical properties and energy absorption efficiency over a wide range of loads when introducing a density gradation in lattices [15]. Tancogne-Dejean et al. studied metallic microlattice materials and demonstrated high specific energy absorption under static and dynamic loading [16]. Spear and Palazotto presented statistical modeling and studied the effect of lattice topology, cell size, cell density, and surface thickness on the mechanical properties of lattice structures.

Furthermore, new lattice configurations with a multitude of base components are being studied, e.g., rod unit-based lattices [17], pyramidal material-filled tube lattices [18]. Lattices offer considerable design degrees of freedom, such as various unit cell geometries and slenderness ratios of trusses, which allows the designer to tailor their design to a specific impact scenario [5]. Broadly speaking, lattices can be tailored to achieve specific objectives, and hence, are titled architected lattices. Recent literature has demonstrated various examples of architected lattices that are potent in energy absorption applications [19,20,21]. In an effort to expand on architected lattices, 2D hierarchical lattices were introduced and studied first due to their simplified geometry and manufacturability when compared to 3D hierarchical lattices. Tsang et al. [22,23] demonstrated a reduction in von Mises stress and an increase in energy absorption for the second order hierarchical 2D tubular structure when compared to the first order tabular model. Additionally, it has been shown that 2D hierarchical honeycombs can provide twice the energy absorption when compared to regular 2D honeycomb structures [22,23]. Other examples of 2D hierarchical structures demonstrating superior mechanical properties can be found in [24,25,26].

In light of recent advancements in additive manufacturing, 3D hierarchical lattices have emerged as a viable potential. Highly efficient compression bearing structures can be obtained using the principle of hierarchical design [27]. It has been reported that the specific stiffness and strength values of hierarchical lattices decrease exponentially with the increase in hierarchical order [28,29]. At nanoscale, experiments revealed theoretical scaling of structural strength and stiffness with relative density for hierarchical nanolattices compared to nonhierarchical nanolattices [28]. Hierarchical metamaterials demonstrated super elastic stretching and large tensile deformations before failure [30]. In addition, experimental results indicated that the mechanical properties of hierarchical lattices were not primarily determined by relative density, unlike those of conventional lattices, but varied with strut slenderness ratios [31]. Moreover, the effects of the hierarchical order, the lattice topology, and the relative density on the piezoelectric effect were investigated, and it was shown that second-order hierarchical metamaterials improve the piezoelectric energy harvesting figure of merit compared with those of first-order counterparts in terms of all reachable relative densities [32].

In order to study the enhanced mechanical properties of hierarchical structures, we focus on studying energy absorption capabilities and the behavior of 3D printed hierarchical lattices by analyzing various unit cell types under low strain rates and loads. By using low strain rates and loads, we can capture the deformation precisely, and also time-dependent material models do not need to be considered. In this work, we first study the effect of introducing a hierarchy to an octet lattice; then, we select three specific unit cells and study the effect of unit cell geometry on the energy absorption, the effect of unit cell orientation on energy absorption, and the effect of changing the lattice members’ cross-section on energy absorption. For our numerical simulations, we select Young’s modulus of TPU 85A as 12 MPa (NinjaTek, D = 2.85 mm). Each specimen is subjected to compression loading up to 50% of the total original lattice height. Finally, we compare the scaling of the stiffness due to the introduction of a hierarchy between simulation results and analytical results.

## 2. Materials and Methods

### 2.1. Generation of Hierarchical Lattices

**Figure 1:**
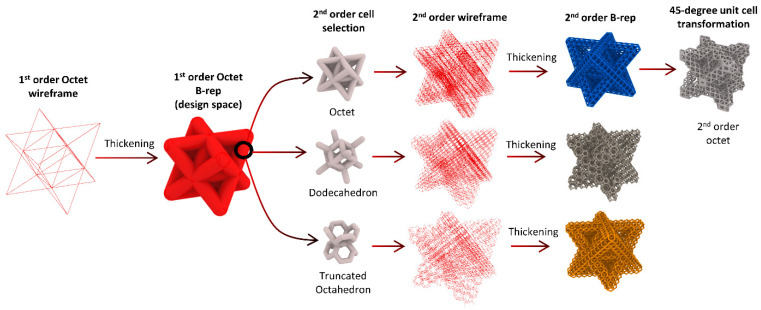
The generation process of hierarchical lattices.

The unit cells of hierarchical lattices are the smallest repeating building blocks in any lattice, and they can be based on different configurations, including but not limited to body-centered cubic (BCC), face-centered cubic (FCC), and simple cubic (SC). We used Rhinoceros 3D V5.0 to design all the lattices in this study. The design process starts with constructing the first order unit cell as a wireframe connecting the nodes of the unit cell. The wireframe of the first order is then given a thickness in the form of a solid circular rod of an adequate diameter to generate a B-rep model. The resultant first order B-rep is utilized as the design space for the second order unit cells to populate. The wireframe of the second order lattice can be converted to a B-rep by thickening the wireframe in the form of a solid circular rod. Moreover, the parametric design tool allows the user to change the orientation of the second order cells relative to the primary plane. The generation process of hierarchical lattices is depicted in Figure 1. The same methodology is followed to generate a second order octet, a second order truncated octahedron, and a second order dodecahedron lattice. The choices of the second order unit cell geometries were made to obtain a diverse set of unit cells with various numbers of nodes and connection links.

**Figure 2:**
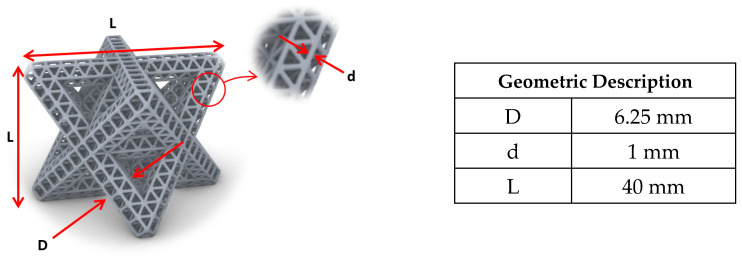
Geometric description of a hierarchical lattice.

The geometric description shown in Figure 2 is kept constant throughout the various designs in this study. We define d as the diameter of the links, L as the major length of the lattice, and D as the major diameter of the lattice, also referred to as the first-order diameter. Furthermore, the number of cells across L was maintained across all the designs in this study.

### 2.2. Finite Element Analysis

The energy absorption characteristics of 3D hierarchical lattices are studied by conducting finite element analyses of compression through a rigid plate pressing against the lattices under low strain rates and loads. The simulations were conducted using ABAQUS (Dassault Systemes Corp, version 2018). The second-order hierarchical lattices possess highly nonlinear geometries and contain many links that may be prone to buckling and kinking [33]. In order to simulate this, nonlinear explicit analysis was used for the second order lattices, whereas implicit analysis was sufficient to simulate the first-order lattices. Instead of using solid continuum elements, beam elements were found to be more suitable to model thin links that exist in hierarchical lattices by providing 6 degrees of freedom per node, while being computationally less expensive when compared to solid continuum elements.

Table 1 summarizes the simulation parameters used for all first- and second-order lattices. For all simulations, to approximate the surface roughness of the printed layers for rubber-like materials, we assumed a hard contact condition for the normal behavior and a frictional contact for the tangential behavior with a coefficient of friction of 0.3 [34]. The normal and tangential contact behaviors are applied between the lattices and the rigid plates as well as for self-contact.

The bottom plate is assigned a fixed boundary condition, while the top plate is displaced 20 mm in the vertical direction to initiate a 50% overall lattice strain. We define the overall lattice strain as the change in lattice height divided by the original height of the lattice. For consistency and comparison, the same approach and boundary conditions were applied to the rest of the second-order lattices in this study.

### 2.3. Material Characterization

**Figure 3:**
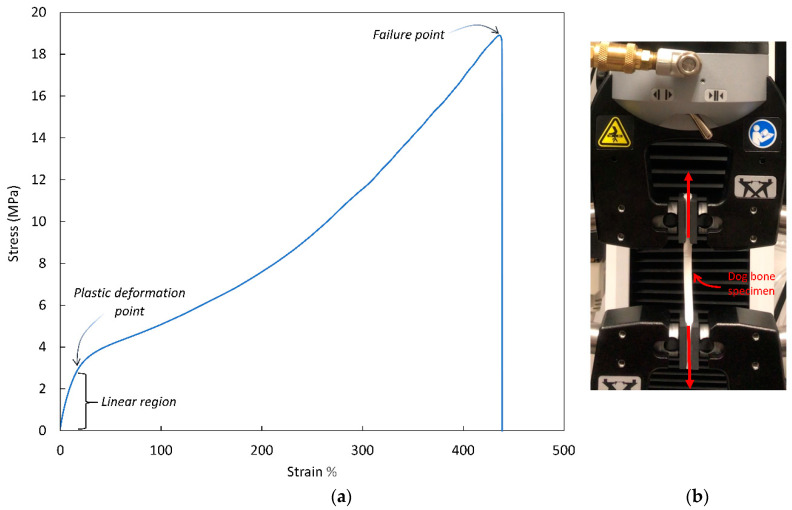
(**a**) Experimental tensile test of a TPU dog bone specimen, (**b**) Instron 3343 tensile testing machine setup.

A material characterization study was carried out on a NinjaFlex thermoplastic polyurethane (TPU) 85A filament (NinjaTek, D = 2.85 mm) by conducting a tensile test of a dog bone specimen. The specimen is loaded in tension until failure. Figure 3 demonstrates the TPU stress–strain curve and its important regions, such as the linear behavior region and the point where plasticity starts. Understanding how the TPU 85A material behaves will provide us with valuable information on what material models can be used in the finite element analysis.

TPU was the candidate of choice due to the mechanical properties it possesses. When compared to other fused deposition materials such as Polylactic acid (PLA) and Acrylonitrile butadiene styrene (ABS), TPU is capable of large deformations under small loads, sustaining high tensile strains before failure, as well as having the capability of being reused after high compression scenarios (50% overall lattice strain). Three dog bone specimens were tested under uniaxial tension to ensure consistency in fabrication and testing. The dog bone specimens were fabricated with a 100% infill and a printing direction parallel to the tensile force. It was observed that for the low-stress region (0 MPa–3 MPa), the behavior of the specimen material could be approximated as linear elastic.

Thus, if the stresses in the lattice are under 3 MPa and nonlinearity exists, one can relate that to geometric nonlinearity effects (buckling, large displacements) and boundary condition nonlinearity effects (contact). The experimental Young’s modulus was found to be 15 MPa, which is close to the manufacturer’s specification of 12 MPa [35]. This variation can be caused by fabrication-related factors such as extruder temperature and nozzle diameter.

## 3. Numerical Simulation

### 3.1. Effect of Hierarchical Order on Energy Absorption

**Figure 4:**
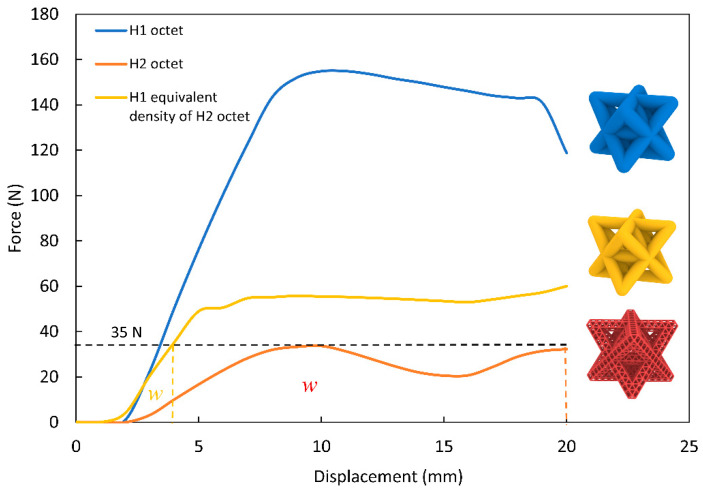
Compression behavior of 1st and 2nd order octet lattices.

In this section, the effect of hierarchical order on energy absorption is investigated. To compare the energy absorption performance independently of density, we define and compare a first-order lattice with the same relative density as a second-order lattice. Figure 4 delineates the relationship between the force and the displacement for three different lattices. From each plot, one can calculate the overall compression stiffness K of each structure as:

(1) $$K=\frac{\Delta F}{\Delta x}$$

and the energy absorbed during the low strain rate (20 mm/s) compression as:

(2) $$W=\int_{0}^{displacement}F\;dx$$

For the purpose of simplifying the terminology in this study, we refer to the first-order lattices as H1, and the second-order lattices as H2. Table 2 summarizes the energy absorption of each lattice at the 35 N load mark using Equation (2).

**Figure 5:**
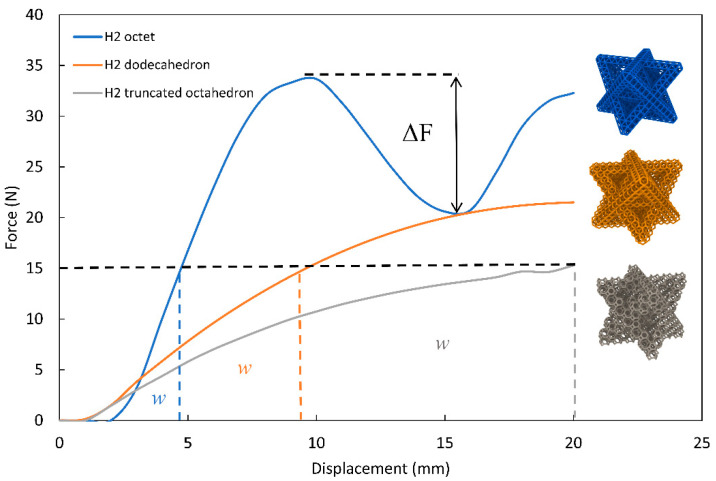
Compression behavior for various 2nd order lattices.

From Figure 4, we can see that the second-order octet has four to five times the energy absorption capacity under low loads, and up to 35 N when compared to the first-order lattices. The second-order octet reaches a plateau regime at an early loading stage (35 N) compared to both first-order octets. This is preferable compared to the linear behavior of the first-order octet, because in the second-order lattice, the energy is dissipated through the buckling of the lattice members, and the friction between the links upon interaction; this can be seen in Figure 5 as ΔF.

The relative density $\rho /\rho_{s}\;$ of the second-order lattice is about 0.29, while the first-order lattice has a density of 0.50. Here, $\rho_{s}\;$ is defined as the density of the solid material, and ρ is the density of the lattice. For single-order lattice structures, energy absorption capacity can be directly associated with lower relative density due to the structure being more deformable, and thus, be more effective at energy absorption. However, in Figure 4, it is observed that the first-order octet with an equivalent density of a second-order octet has four to five times less energy absorption capacity than the second-order octet.

### 3.2. Effect of Unit Cell Geometry on Energy Absorption

In this section, we study the effect of the unit cell geometry on the energy absorption capacity of hierarchical lattices; we analyze three different unit cells under compression and compare the energy absorbed up to a specific force. Figure 5 demonstrates the effect of unit cell geometry on energy absorption. By varying the second order unit cell geometry, we can alter the compression behavior of the lattice. For instance, the second order octet demonstrated a pronounced buckling between the 10 mm and the 15 mm displacement marks, shown as ΔF in Figure 5. The second-order dodecahedron and truncated octahedron exhibited a foam-like behavior [36], with a smooth continuous nonlinear deformation throughout the 50% strain-imposed boundary condition. Comparing the energy absorption capacity of each lattice, we see that the truncated octahedron lattice curve covers the largest area at the 15 N mark.

The volumetric energy absorption efficiency η, which is expressed as the ratio of the area under the force–displacement curve divided by the maximum force achieved up to a given displacement, can be used to compare different lattices [5]. The energy absorption efficiency is calculated using:

(3) $$\eta =\frac{\int_{0}^{\varepsilon }F\;d\varepsilon }{Max\left(F\left(\varepsilon \right)\right)}$$

where *F* is the force, and ε is the overall lattice strain. The results demonstrated in Table 3 show that the volumetric energy efficiency decreases for lattices with the delayed flat plateau region. The volumetric energy efficiency of the second-order truncated octahedron lattice is higher than that of the dodecahedron lattice. Among the three different unit cells tested, we conclude that the truncated octahedron lattice is the superior option for energy absorption applications under the prescribed 15 N load condition. In comparison, the octet lattice can serve as a viable option for higher load scenarios due to its higher stiffness.

**Figure 6:**
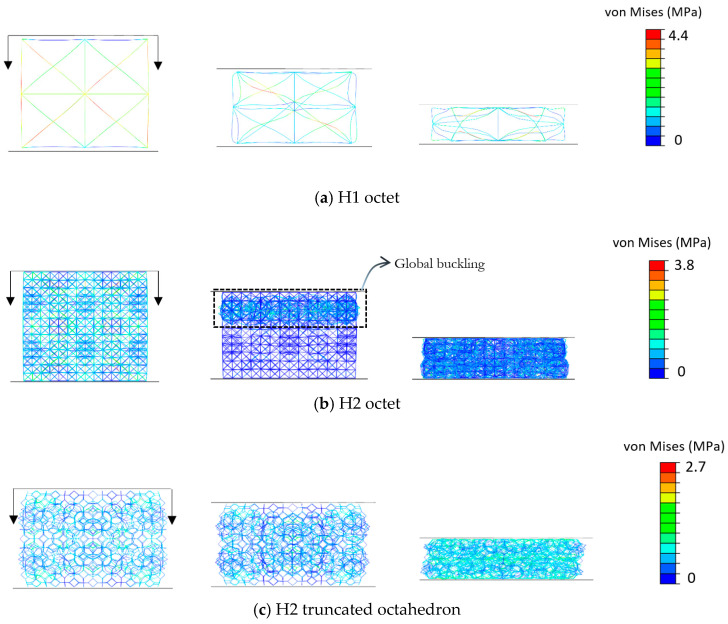

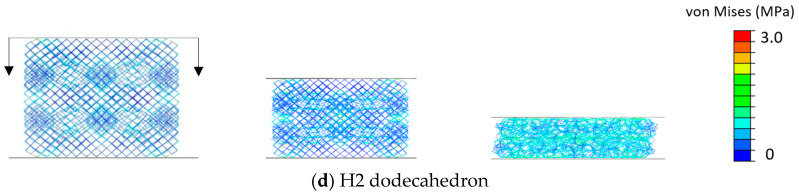
FEA von Mises stress contour plots for various lattices under compression of 50% overall lattice strain. (**a**) first order octet lattice, (**b**) second order octet lattice, (**c**) second order truncated octahedron lattice, (**d**) second order dodecahedron.

Figure 6 displays the compression behavior of each of the H1 and H2 lattices. The highly stressed H1 octet with a maximum stress of 4.4 MPa corresponds to the lattice with the lowest energy absorption capacity in Figure 5. Similarly, the lattice with the lowest von Mises stress (H2 truncated octahedron) corresponds to the lattice with the highest energy absorption capacity. The maximum von Mises stresses for the H1 octet, H2 octet, H2 dodecahedron, and H2 truncated octahedron are 4.4 MPa, 3.8 MPa, 3 MPa, and 2.8 MPa, respectively.

It is important to note that the highest stress occurs at very few links (5% < of total lattice links) in the lattice, and the von Mises stresses at other regions of the lattices are much smaller (10–15% of maximum von Mises stress) than the highest local von Mises stress, thus experiencing a very negligible plastic deformation (5% < of lattice links experience plastic deformation). This can be seen from the colored stress contour plots in Figure 6; the results also strengthen the linear elastic model assumption used in the study. Moreover, Figure 6b above demonstrates a global buckling in the lattice, which explains the ΔF (drop) observed in Figure 5 for the second-order octet lattice between the 10 mm and 15 mm displacement marks. We define global buckling as a buckling that causes a section of the lattice to collapse, unlike local buckling that occurs locally in lattice members without any global effect.

### 3.3. Effect of Unit Cell Orientation on Energy Absorption

**Figure 7:**
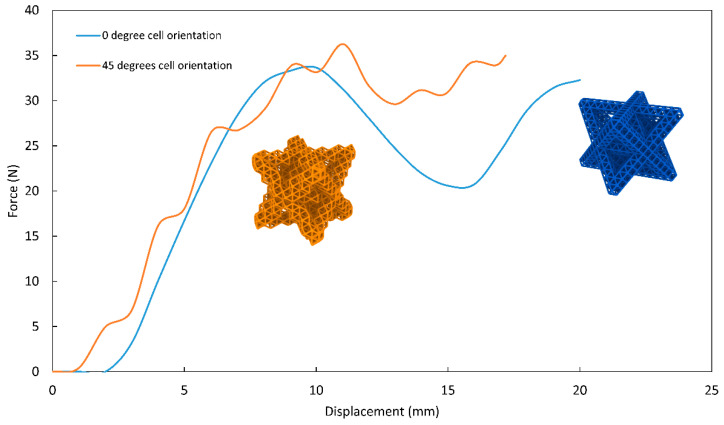
Effect of unit cell orientation on the compression behavior and the energy absorption of H2 octet.

In Figure 7, the force–displacement plot of the two second-order octet lattices is presented, which would enable us to study the compression behavior as well as the absorbed energy. One lattice had second-order cells making a 0-degree angle with the XY plane; the second lattice had second-order cells making a 45-degree angle with the XY plane. Here, we define the XY plane as the horizontal flat plane. The behavior of the second order lattice with respect to the orientation of the unit cell is investigated under compression. The 45-degree-oriented second-order lattice displayed less pronounced global buckling behavior between the 10 mm and 15 mm displacement marks.

However, a higher local buckling effect is demonstrated as ripples along the force–displacement curve. The overall energy absorption capacity of the 45-degree-oriented second-order octet lattice is about 20% greater than its non-oriented counterpart.

### 3.4. Effect of Cross-Sectional Geometry of Lattice Members on Energy Absorption

**Figure 8:**
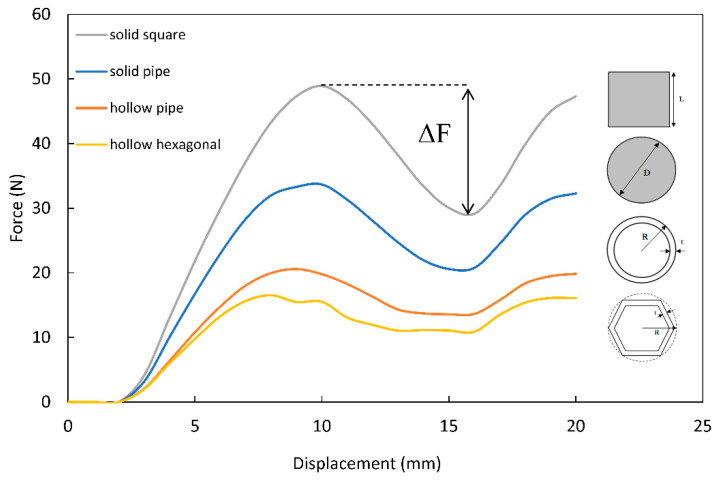
Effect of lattice members’ cross-sectional geometry on energy absorption.

Figure 8 depicts the effect of changing the cross-sectional geometry of the lattice members on the force–displacement curve for a solid square, solid circular, hollow circular, to hollow hexagonal on a second order octet lattice. The geometric description of each cross-section can be found in Table 4. The results show that for the hollow cross-sections, the buckling occurs consistently, with no abrupt changes in force; this can be seen in the region between the 10 mm to the 18 mm displacement marks. In comparison, the solid counterparts such as the solid circular pipe, and the solid square, exhibited an abrupt change in the transmitted force, demonstrated by ΔF in the plot.

The change in force ΔF can be interpreted as a change in the momentum rate, resulting in unwanted shocks, which is not preferable in energy absorption structural designs. The second-order octet lattice with hollow members recorded the highest energy absorption efficiency; this is because the hollow members are more prone to buckling when compared to their solid counterparts, and thus, the lattice will absorb more energy upon collapsing. Table 4 summarizes the maximum magnitude of ΔF and the volumetric energy absorption efficiency with respect to the cross-sectional geometry of the lattice members.

### 3.5. Comparing with Analytical Solution of an Octet Lattice

The analytical solutions for the first-order and second-order octet lattices were investigated and compared to the numerical results discussed in Section 3.1. The effective relative density of a second-order octet lattice is obtained using the following equations [28,36],

(4) $${{\bar{\rho }}^{\left(Octet\right)}}_{2}=\frac{36Q-92}{Q^{3}}\left[\frac{25\sqrt{2}\pi }{16}{\left(\frac{d_{1}}{l_{1}}\right)}^{2}-\left(5.922\right){\left(\frac{d_{1}}{l_{1}}\right)}^{3}\right]$$

(5) $$\bar{\rho_{1}}=\frac{m}{V}$$

(6) $$E_{2nd\;order}=E_{s}C{\left(\frac{{\bar{\rho }}^{\left(Octet\right)}2_{\;}}{\rho_{s}}\right)}^{2}$$

(7) $$E_{1st\;order}=E_{s}C\left(\frac{\bar{\rho_{1}}}{5}\right)$$

All the parameters used in the analytical solutions are summarized in Table 5. The relative density of the first order is obtained through the conventional way (see Equation (5)). Using Equations (6) and (7), we can obtain the effective elastic modulus for both the first- and second-order octet lattices. For both equations, *C* is a proportionality constant which is close to unity for $\frac{\rho }{\rho_{s}}<0.3$ [36], $E_{s}$ is elastic modulus of the solid constituent material, and $\rho_{s}$ is the density of the solid constituent material.

We compare the ratio $\frac{E_{1}}{E_{2}}=\frac{E_{1st\;order}}{E_{2nd\;order}}$ of the FEA solution with the analytical solution. The comparison allows us to validate the scaling of the effective material properties with respect to the order of hierarchy. $E_{1}\;\mathrm{and}\;E_{2}$ values for the analytical solution are obtained from Equations (6) and (7) respectively, while $E_{1}\;\mathrm{and}\;E_{2}$ values are obtained from the FEA simulation solution. For the FEA results, the ratio of $E_{1}$/$E_{2}$ is 9.26, while the analytical solution yielded a $E_{1}$/$E_{2}$ ratio of 8.57. The difference is within 7.5%, which can be attributed to approximations used in the contact model as well as the material model in the simulation.

## 4. Conclusions

This study investigated the design and energy absorption characteristics of 3D hierarchical lattices to create high-efficiency energy absorption designs. The influences of hierarchy order, unit cell geometry, cell orientation and cell cross-sectional geometry in the lattice structures were studied. We conclude that by introducing a hierarchy, the energy absorption performance of the lattice increases up to four to five times under low loads and strain rates. Through hierarchical arrangements, the number of lattice links that are prone to buckling increases, which facilitates mechanisms for energy absorption. We found that lowering the relative density by itself did not provide the best energy absorption capacity, but tailoring the second-order unit cells improved the energy absorption capacity under certain conditions. The second-order truncated octahedron lattice demonstrated the best energy absorption performance under low loads when compared to the octet and the dodecahedron second-order lattices. Changing the second-order unit cells’ orientation by tilting the cells 45 degrees with respect to the base XY plane caused a moderate increase in stiffness, and a 20% increase in energy absorption capacity. In addition, hollow members performed better than their solid counterparts in terms of energy absorption; reducing the material in the links reduces their stiffnesses, and therefore, facilitates the buckling behavior to absorb more energy. The findings of the study to understand the mechanical response of hierarchical lattices will help facilitate the design of superior energy absorbing structures.

We believe that by implementing hierarchical lattices in the design of athletic shoes, football helmet pads, and other impact protection gear, valuable functional enhancements in energy absorption can be achieved. The future work of this study aims at implementing topology optimization algorithms on hierarchical lattices in order to obtain an optimized energy absorption lattice for a specific load scenario. In addition, since most energy absorption applications are associated with high-velocity impacts, such as protective equipment in sports and crashworthiness applications, the dynamic behavior of the hierarchical structures could be explored further with high strain rates.

## Figures and Tables

**Table 1:** Simulation parameters for all first- and second-order lattices.

Parameter	1st Order	2nd Order
Material properties	TPU 85A, E = 12 MPa [35], density 1.12 g/cc	
Solid member diameter	6.25 mm	1 mm
Impact velocity	20 mm/s	
Mesh element type	B31 (2-node linear beam element)	B32 (3-node quadratic beam element)
Number of elements	396	28,752
Analysis type	Implicit	Explicit
Loading condition	50% overall lattice strain	

**Table 2:** Effect of introducing a hierarchy on the energy absorption.

Lattice Design	Energy Absorbed @ 35 N
First order octet lattice	0.06 J
Second order octet lattice	0.28 J
First order octet with relative density as second order octet	0.07 J

**Table 3:** Energy absorption and volumetric energy absorption efficiency of various 2nd order hierarchical lattices.

Unit Cell Geometry	Energy Absorbed @ 15 N	Volumetric Efficiency Factor η @ 15 N
Octet	0.03 J	0.03
Dodecahedron	0.08 J	0.12
Truncated octahedron	0.18 J	0.30

**Table 4:** Summary of stiffness, magnitude of ΔF, and volumetric energy absorption efficiency 𝜼 for various member with different cross-sectional geometries of H2 octet lattice.

Cross-Sectional Geometry	Dimensions	Stiffness K	Max Magnitudeof the ΔF	Volumetric Energy Absorption Efficiency η @ 50% Strain (20 mm Displacement)
Solid square	L = 1 mm	8.8 N/mm	20 N	0.33
Solid circular	D = 1 mm	6.6 N/mm	12 N	0.35
Hollow circular	R = 0.5 mm t = 0.125 mm	4 N/mm	5 N	0.40
Hollow hexagonal	R = 0.5 mm t = 0.125 mm	4 N/mm	5 N	0.40

**Table 5:** Analytical parameters and results of the H1 and H2 octet.

Symbol	Description	Value
$d_{1}$	Diameter of second order member	1 mm
$l_{1}$	Length of second order member	4.5 mm
Q	Number of unit cells across L/2	7
$\rho_{s}$	Density of solid material	1.2 g/cc
$\bar{\rho_{1}}$	Density of first order lattice	0.5 g/cc
${{\bar{\rho }}^{\left(Octet\right)}}_{2}$	Density of second order lattice	0.13 g/cc
$E_{s}$	Elastic modulus of solid material	12 MPa
$E_{1st\;order}$	Effective elastic modulus of first order lattice	1.2 MPa
$E_{2nd\;order}$	Effective elastic modulus of second order lattice	0.14 MPa

## Data Availability

The data presented in this study are available on request from the corresponding author.
